# Insights into E-Cadherin Impairment in *CDH1*-Unaltered Invasive Lobular Carcinoma: A Comprehensive Bioinformatic Study

**DOI:** 10.3390/ijms25168961

**Published:** 2024-08-17

**Authors:** Shiro Uchida, Takashi Sugino

**Affiliations:** 1 Division of Diagnostic Pathology, Kikuna Memorial Hospital, 4-4-27, Kikuna, Kohoku-ku, Yokohama 222-0011, Japan; 2 Division of Pathology, Shizuoka Cancer Center, Shizuoka 411-8777, Japan; t.sugino@scchr.jp; 3 Department of Human Pathology, Juntendo University School of Medicine, Tokyo 113-8421, Japan

**Keywords:** breast, invasive lobular carcinoma, *CDH1*, E-cadherin

## Abstract

Invasive lobular carcinoma exhibits unique morphological features frequently associated with alterations in *CDH1*. Although some studies have identified abnormalities in adhesion factors other than E-cadherin, the molecular mechanisms underlying E-cadherin abnormalities in *CDH1*-unaltered invasive lobular carcinoma remain poorly understood. In this study, we investigated the molecular underpinnings of E-cadherin dysregulation in invasive lobular carcinoma in the absence of *CDH1* gene alterations, using comprehensive bioinformatic analyses. We conducted a comparative study of *CDH1*-mutated and non-mutated invasive lobular carcinoma and evaluated the differences in mRNA levels, reverse-phase protein array, methylation, and miRNAs. We observed that invasive lobular carcinoma cases without *CDH1* alterations exhibited a significantly higher incidence of the Claudin-low subtype (*p* < 0.01). The results of the reverse-phase protein array indicate no significant difference in E-cadherin expression between *CDH1*-mutated and non-mutated cases. Therefore, abnormalities in E-cadherin production also exist in *CDH1* non-mutated invasive lobular carcinoma. Considering that there are no differences in mRNA levels and methylation status, post-translational modifications are the most plausible explanation for the same. Hence, future studies should focus on elucidating the mechanism underlying E-cadherin inactivation via post-translational modifications in *CDH1* non-mutated invasive lobular carcinoma.

## 1. Introduction

Invasive lobular carcinoma (ILC) is one of the most frequently diagnosed specialized forms of breast cancer, accounting for approximately 5%–15% of all invasive breast cancer cases [1]. It is generally characterized by tumor cells that have lost their connectivity, and its unique histological appearance results from the inactivation of E-cadherin, encoded by the *CDH1* gene [1]. *CDH1* is located on the long arm of chromosome 16 (16q22.1) and is expressed in epithelial tissues. E-cadherin is a transmembrane glycoprotein that promotes calcium-dependent intercellular adhesion [2,3]. It bears extracellular, transmembrane, and intracellular domains, with the extracellular domain largely mediating intercellular adhesion and the intracellular domain being responsible for linking the protein to the cytoskeleton via α-, β-, and γ-catenin [4]. Previous studies have found abnormalities in the *CDH1* coding sequence in 54% and 65% of ILC cases [5,6]. Abnormal *CDH1* promoter methylation has also been frequently reported [7,8]. Nevertheless, the question of whether epigenetic gene silencing is responsible for the loss of *CDH1* expression in ILC remains controversial [1,9]. In previous reports, immunohistochemical analysis showed that approximately 90% of ILC cases were E-cadherin-negative [10,11], i.e., a higher proportion compared to cases of *CDH1* genetic abnormalities, suggesting that even in *CDH1*-unaltered ILC, E-cadherin production can be impaired. Other studies have shown that mutations in α-catenin (encoded by the *CTNNA1* gene), which forms the cadherin–catenin complex that is important for intercellular adhesion, may underlie the lobular carcinoma phenotype even in the absence of *CDH1* mutations and the presence of normal *CDH1* expression [12]. Furthermore, a recent study examined the abnormal inactivation of other adhesion factors, such as CTNND1 and AXIN2, in ILC in the absence of CDH1 genetic alterations [13]. Overall, it is suggested that E-cadherin function can be impaired through various mechanisms in *CDH1*-unaltered ILC. However, studies focusing on *CDH1*-unaltered ILC are scarce, and several aspects regarding its clinicopathological (age, site, menopausal status, T factor, N factor, and tumor grade) and molecular characteristics (tumor mutational burden and co-mutated genes) remain unknown.

In the present study, we used bioinformatics tools, such as BioPortal, to analyze the clinicopathological features of ILC in the presence (*CDH1*-altered ILC) and absence (*CDH1*-unaltered ILC) of *CDH1* alterations, utilizing datasets from The Cancer Genome Atlas (TCGA) and the Molecular Taxonomy of Breast Cancer International Consortium (METABRIC). Additionally, LinkedOmics was used to analyze the differences at the multi-omics level between *CDH1*-mutated and *CDH1* non-mutated ILC. 

## 2. Results

### 2.1. Clinicopathological Features, Intrinsic Subtype, and Tumor Mutational Burden of CDH1-Altered and -Unaltered ILC

We identified 110 samples of *CDH1*-altered ILC among the 201 ILC samples (110/201, 54.7%) in TCGA dataset and 91 samples among the 192 ILC samples (91/192, 47.4%) in the METABRIC dataset, yielding a total of 201 *CDH1*-altered ILC cases. *CDH1* alterations included 13 missense (6.5%), 5 in-frame (2.5%), 23 splice-site (11.4%), 147 truncating (73.1%), 4 homozygous deletion-with-mutation (2.0%), 7 homozygous deletion-only (3.5%), and 2 structural variant (fusion) (1.0%) mutations (Table 1). All of the gene mutations were annotated using OncoKB [14]. Two structural variants (fusions), *CDH1*–*NADK2* and *CDH1*–*VPS50*, were also identified. Annotations in OncoKB indicated that both were likely oncogenic, demonstrating a loss of *CDH1* function.

**Figure 1:**
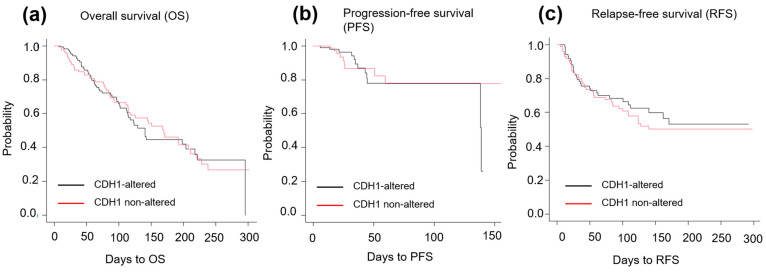
Overall survival (OS), progression-free survival (PFS), and relapse-free survival (RFS) in *CDH1*-altered and -unaltered ILC. (**a**) No significant difference in OS was observed between *CDH1*-altered (n = 181) and -unaltered (n = 166) ILC (*p* = 0.97). (**b**) No significant difference in PFS was observed between *CDH1*-altered (n = 110) and -unaltered (n = 91) ILC (*p* = 0.97). (**c**) No significant difference in RFS was observed between *CDH1*-altered (n = 86) and -unaltered (n = 98) ILC (*p* = 0.57).

No significant differences were observed in clinicopathological features between *CDH1*-altered and -unaltered ILC in terms of age, site of origin, menopausal status, T factor, N factor, and tumor grade (Table 2). No significant differences in the overall survival, disease-free status, or relapse-free status were identified between the groups either (Figure 1a–c).

Tumor mutational burden (TMB) was not significantly different between the *CDH1*-altered and -unaltered groups (*p* = 0.05); 84.1% of the *CDH1*-altered group had a low TMB and 15.9% had a high TMB, whereas 88.3% of the *CDH1*-unaltered group had a low TMB and 11.7% had a high TMB (Table 2). Among the intrinsic subtypes, a significant prevalence of the Claudin-low (CL) subtype was observed in *CDH1*-unaltered ILC in the METABRIC dataset. No significant difference between the two groups was found in the TCGA analysis (Table 3).

### 2.2. Genomic Landscape of Somatic Mutations and Comparison of Somatic Mutations and Amplification between CDH1-Altered and -Unaltered ILC

**Figure 2:**
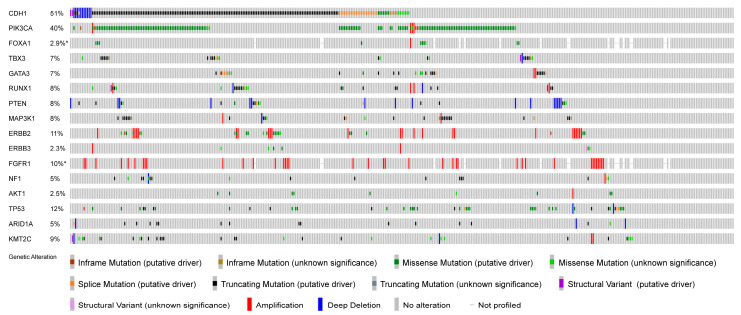
The genomic landscape of ILC-integrated TCGA and METABRIC datasets. The Oncoprint showing the frequency of genes co-mutated in ILC and copy number alterations. Each column represents a single sample. The frequency of alterations is shown on the left. Multiple genes with altered mutation frequencies, including ILC-related genes, are displayed.

Oncoplots summarizing all gene names, the presence/absence of mutations, mutation type, mutation frequency, and amplification/deletion of *ERBB2*, *ERBB3*, and *FGFR1* for each group within *CDH1*-altered and -unaltered ILC are shown in Figure 2. The results of detailed statistical analyses are summarized in Table S1. *PIK3CA*, *RUNX1*, and *ERBB2* mutation frequencies were significantly lower in *CDH1*-unaltered ILC than in *CDH1*-altered ILC (*p* < 0.05). However, there were no significant differences in other gene mutations or in the amplification of *ERBB2*, *ERBB3*, or *FGFR1*.

### 2.3. Identification of Differences in CDH1 mRNA, Protein, Methylation, and miRNA (hsa-mir-676) Levels between CDH1-Mutant and Non-Mutant Groups Using LinkedOmics

**Figure 3:**
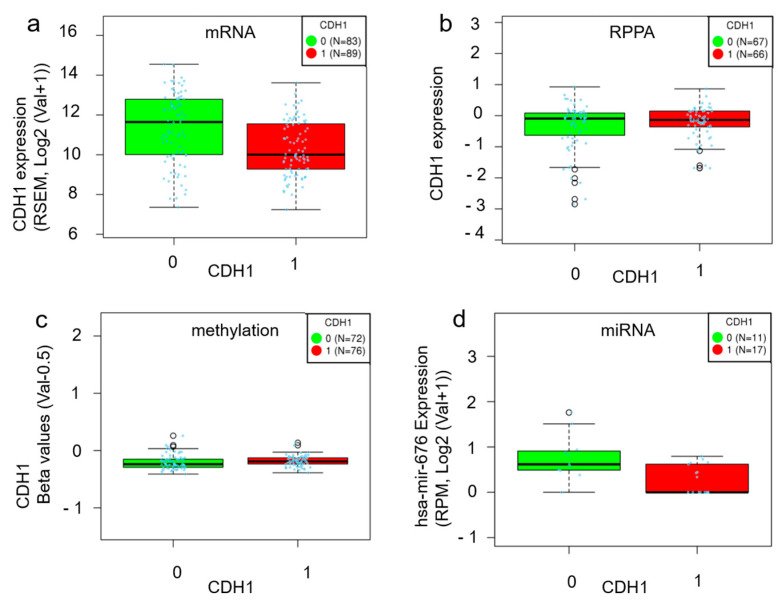
A comparison of *CDH1* mRNA, protein, methylation, and hsa-mir-676 levels between *CDH1*-mutated and non-mutated ILC. (**a**) No significant difference in *CDH1* mRNA levels was observed between *CDH1*-altered and -unaltered ILC (*p* < 0.01, false discovery rate (FDR) = 0.37). The Y-axis represents mRNA expression. RSEM: RNA-Seq by Expectation–Maximization. (**b**) No significant difference in *CDH1* protein levels was observed between *CDH1*-mutated and non-mutated ILC (*p* = 0.64, FDR = 0.81). The Y-axis represents reverse-phase protein array (RPPA) expression. (**c**) No significant difference in *CDH1* methylation levels was observed between *CDH1*-mutated and non-mutated ILC (*p* = 0.04, FDR = 0.44). The Y-axis represents β values (Val-0.5). (**d**) No significant difference in hsa-mir-676 levels was observed between *CDH1*-mutated and non-mutated ILC (*p* = 0.01, FDR = 0.99). The Y-axis represents expression (RPM, Log2(Val + 1)). RPM: Reads Per Million. The figure shows 0 as *CDH1* non-mutated ILC and 1 as *CDH1*-mutated ILC.

LinkedOmics analysis revealed no significant differences in the levels of *CDH1* mRNA, protein (E-cadherin), methylation, or hsa-mir-676 between *CDH1*-mutant and non-mutant ILC (Figure 3). Among the miRNAs, hsa-mir-676 was the most highly expressed in *CDH1* non-mutant ILC; however, no significant differences were identified in RNA-seq, reverse-phase protein array (RPPA), methylation, or miRNA analyses. In summary, there were no significant changes in *CDH1* mRNA, protein, or methylation levels in the presence or absence of *CDH1* mutations, and no miRNAs were significantly upregulated in *CDH1* non-mutant ILC.

### 2.4. Gene Set Enrichment Analysis (GSEA) of Gene Ontology Annotations

**Figure 4:**
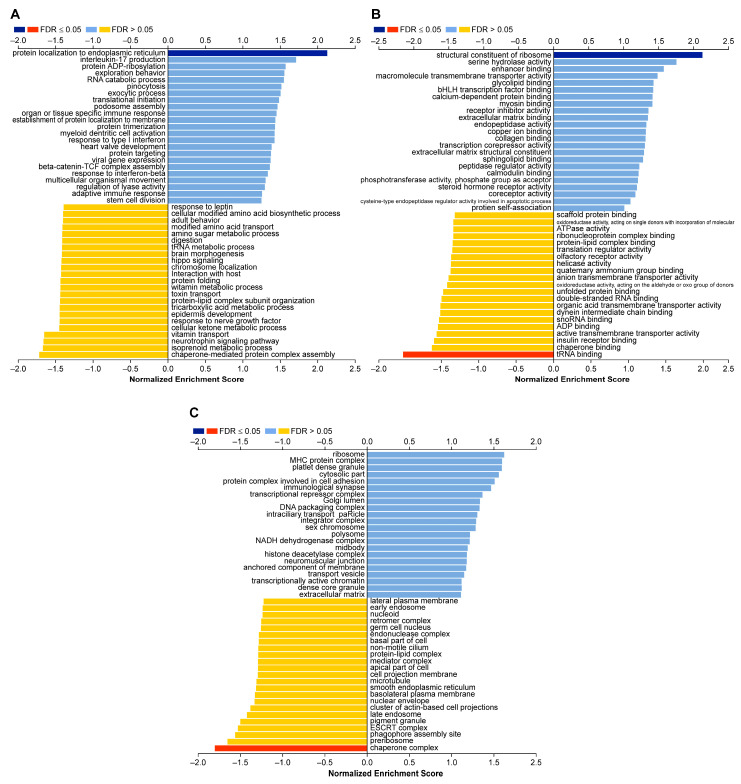
Gene Ontology (GO) annotation analyses by LinkedOmics using GSEA methods. (**A**) Bar chart of biological process. Protein localization in endoplasmic reticulum showed significant differences. Despite displaying FDR > 0.05, chaperone-mediated protein complex assembly and protein folding were also recognized as important GO terms. (**B**) Bar chart of molecular function. tRNA showed significant differences. (**C**) Bar chart of cellular components. Chaperone complex showed significant differences.

Via GSEA, we identified several key Gene Ontology (GO) terms, with protein localization in the endoplasmic reticulum showing significant enrichment. Although the false discovery rates (FDRs) exceeded 0.05, chaperone-mediated protein complex assembly and protein folding were recognized as important biological processes (Figure 4A). Furthermore, the structural constitution of ribosomes and tRNA binding were determined to be crucial for molecular function (Figure 4B). It is important to note that the chaperone complex remains an essential and major cellular process (Figure 4C). In summary, GSEA revealed significant differences in various functions related to protein synthesis processes between CDH1-mutated and CDH1 non-mutated ILC.

## 3. Discussion

We used datasets from TCGA and the METABRIC to examine *CDH1* gene alterations in ILC. We classified ILC into *CDH1*-altered and -unaltered groups and compared their clinicopathological characteristics and associated genetic mutations. We found that *CDH1* alterations were present in 51.1% (201/393) of ILC cases, with truncating mutations comprising 73.1% (134/201) of these alterations, making them the most prevalent mutation type observed. The observed *CDH1*-alteration frequency and the high number of truncating mutations were generally consistent with previous reports [5,6,9]. Two fusion genes, *CDH1*–*NADK2* and *CDH1*–*VPS50*, were identified in this study. Both were annotated as likely oncogenic by OncoKB, suggesting a loss of *CDH1* function. When comparing *CDH1*-altered and -unaltered ILC, *PIK3CA*, *RUNX1*, and *ERBB2* mutation frequencies were significantly higher in *CDH1*-altered ILC. This could be attributed to the fact that *PIK3CA* and *RUNX1* mutations are commonly observed in luminal-type breast cancer; in the intrinsic subtype, *CDH1*-altered ILC exhibited a higher proportion of luminal-type cases. This may explain the high occurrence of these genetic mutations [15]. *ERBB2* mutations are significantly enriched in *CDH1* mutations and are considered common in *CDH1*-altered ILC [16,17,18].

In the METABRIC dataset, *CDH1*-unaltered ILC had a higher proportion of CL intrinsic subtype cases compared to *CDH1*-altered ILC. The CL type is characterized by low expression of tight junction proteins and adhesion proteins (Claudins 3, 4, and 7 and E-cadherin) and high expression of mesenchymal, extracellular matrix, and epithelial–mesenchymal transition (EMT) proteins, such as vimentin, SNAI 1/2, Twist 1/2, and ZEB 1/2 [19]. Previous reports have shown that metaplastic carcinoma and invasive ductal carcinoma are common in CL, but ILC has also been reported to occur in approximately 4% of cases [19,20]. Generally, EMT-related genes such as *TWIST* and *SNAIL* are known to suppress E-cadherin expression. However, although EMT typically occurs in the final stages of tumor progression, in ILC, the E-cadherin level decreases from the early stages of development. It has been reported that the low E-cadherin level in ILC is not associated with EMT [5].

With respect to the prognostic value of reduced E-cadherin levels in breast cancer, previous studies found that these promoted tumor invasion and metastasis [3,21]; as a result, overall survival and disease-free survival duration were significantly shorter and correlated with tumor histological grade, TNM stage, tumor size, and the presence of lymph node metastasis [22,23,24]. Although we anticipated that the presence or absence of *CDH1* mutations would result in differences at the protein levels, interestingly, LinkedOmics analysis revealed no significant differences in E-cadherin levels when comparing *CDH1*-mutated with non-mutated ILC. These findings indicate that in *CDH1* non-mutated ILC, although there are no mutations in the *CDH1* gene, E-cadherin expression is reduced, suggesting that abnormalities in E-cadherin protein synthesis may occur even in *CDH1* non-mutated ILC.

The previous literature has identified five mechanisms of E-cadherin inactivation in breast cancer: (i) mutation, (ii) a loss of heterozygosity, (iii) promoter methylation, (iv) transcriptional repression, and (v) post-translational modifications (such as aberrant glycosylation) [25]. The existence of N-terminal-deficient impaired E-cadherins has recently been proposed. Although the mechanism underlying the production of N-terminal-deficient E-cadherins is still unknown, post-translational modifications are thought to be involved [26]. The mechanism underlying E-cadherin inactivation in *CDH1* non-mutated ILC is unlikely to involve promoter methylation or transcriptional repression, as LinkedOmics analysis revealed no differences in mRNA or methylation levels. Additionally, no miRNAs inhibiting *CDH1* expression were identified. In contrast, the mechanisms of E-cadherin inactivation, such as post-translational modifications (e.g., aberrant glycosylation) and N-terminal-deficient E-cadherin production, are independent of genetic alterations, promoter methylation, and transcriptional repression. Therefore, these mechanisms could be plausible contributors to E-cadherin dysregulation in *CDH1* non-mutated ILC. In addition, GSEA detected GO terms associated with protein production (i.e., protein localization to the endoplasmic reticulum, the structural constitution of ribosomes, ribosome activity, tRNA binding, and the chaperone complex), which supports the hypothesis of abnormalities in protein production in *CDH1* non-mutated ILC. Post-translational modifications such as O- and N-glycosylation are reportedly important for E-cadherin production [27,28] as well as its folding, transport, and stability at the membrane [29,30,31,32]. E-cadherin deglycosylation at Asn-633 has been shown to result in its degradation via the endoplasmic reticulum-associated degradation mechanism in the absence of correct folding [31]. Such post-translational modifications have been suggested for E-cadherin in *CDH1* non-mutated ILC. As a result of these abnormalities, GO terms related to protein production may have been detected. Regarding N-terminal-deficient E-cadherin production, it is currently considered that incomplete E-cadherin lacking the N-terminus is produced via post-translational modifications involving enzymatic cleavage by matrix metalloproteinases (MMPs), a disintegrin and metalloproteinases (ADAMs), and neutrophil elastase [26].

The findings of this study suggest that post-translational modifications (such as aberrant glycosylation) and N-terminal-deficient E-cadherin production play a significant role in the pathogenesis of *CDH1*-unaltered ILC. Currently, the mechanisms underlying E-cadherin inactivation via post-translational modifications have not been elucidated, necessitating further research in this area. This study presented an important limitation. It exclusively used bioinformatic approaches and considered abnormal E-cadherin production through impaired protein production processes and post-translational modifications as a possible molecular mechanism of *CDH1*-unaltered ILC, but did not validate the findings using human tissues or cultured cells from breast lobular carcinoma. In the future, it will be necessary to experimentally investigate whether glycosylation abnormalities actually occur in ILC cell lines and human specimens of ILC and whether proteolytic enzymes such as MMPs and ADAMs are indeed involved in the production of N-terminal-deficient E-cadherin.

## 4. Materials and Methods

### 4.1. Data Collection of CDH1-Altered and -Unaltered ILC

**Figure 5:**
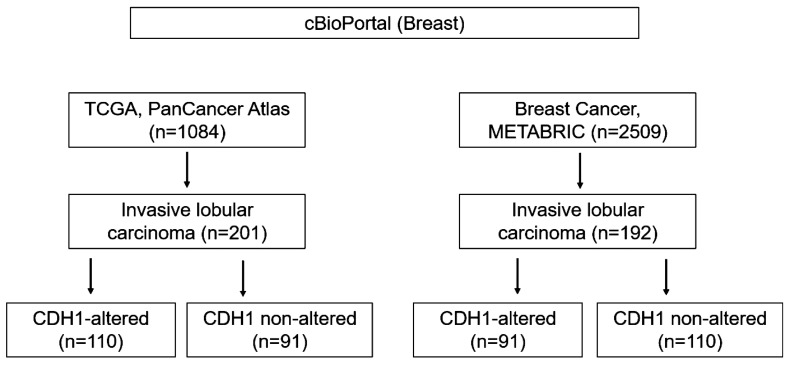
Flowchart of case selection.

Genomic and clinical data were collected from breast invasive lobular carcinoma samples using cBioPortal. Specifically, data from TCGA PanCancer Atlas dataset (Breast Invasive Carcinoma) [33] and the METABRIC dataset (breast cancer) [34,35] (n = 201 and n = 192, respectively) were obtained. Next, we categorized the cases into *CDH1*-altered and -unaltered ILC. *CDH1* alterations were considered to include structural variants (fusions), copy number alterations (homozygous deletions), and mutations. For TCGA and the METABRIC, *CDH1*-altered (n = 110 and n = 91) and -unaltered (n = 91 and n = 101) ILC cases were identified, respectively, resulting in a total of 201 *CDH1*-altered and 192 *CDH1*-unaltered ILC cases. A detailed case selection flowchart is shown in Figure 5.

### 4.2. Comparison of Clinicopathological Features and Molecular Subtypes between CDH1-Altered and -Unaltered ILC

For ILC samples, the age (mean), sex distribution, site of onset, T factor, N factor, tumor grade, and molecular subtype were obtained from cBioPortal (New York, NY, USA). Where information was not available, it was designated as “N/A” and excluded from percentage calculations. However, no tumor grade information was available for TCGA dataset. In addition, the overall survival, disease-free status, and relapse-free status were analyzed. Overall survival analysis was performed by integrating data from TCGA and the METABRIC, with data from TCGA being used only for disease-free status and those from the METABRIC only for relapse-free status. In TCGA dataset, intrinsic subtypes were determined using the PAM50 multi-gene assay and classified into five categories: luminal A, luminal B, Her-2 enriched, basal-like, and normal-like. Conversely, in the METABRIC dataset, classification using the PAM50 assay was expanded to include the CL subtype, which allowed for categorization into six distinct types.

### 4.3. TMB Estimation

TMB is a measure of the total number of mutations per megabase (Mb) of tumor tissue. It can also be interpreted as the mutation density in tumor genes, defined as the average number of mutations in the tumor genome, including the total number of coding sequence errors, base substitutions, insertions, or deletions. This information was obtained from two datasets. The samples were classified as TMB-high if they had ≥10 mutations per Mb (mut/Mb) and TMB-low if they had <10 mut/Mb, as described previously [36]. We compared the TMB for each cancer type between the *CDH1*-altered and -unaltered ILC groups, as well as the TMB low-to-high ratio.

### 4.4. CDH1 Alteration Analysis and Comparison of Mutation and Amplification Frequency of CDH1-Related Genes between CDH1-Altered and -Unaltered ILC

We used cBioPortal to investigate ILC and subsequent genetic alterations. Genes closely related to ILC were selected from previous studies [9,37], and the frequency of each gene mutation was compared between *CDH1*-altered and -unaltered ILC: *PIK3CA*, *FOXA1*, *TBX3*, *GATA3*, *RUNX1*, *PTEN*, *MAP3K1*, *ERBB2*, *ERBB3*, *FGFR1*, *NF1*, *AKT1*, *TP53*, *ARID1A*, and *KMT2C*. We also investigated *ERBB2*, *ERBB3*, and *FGFR1* amplification. The detected genetic alterations were visualized using Oncoprinter on the cBioPortal.

### 4.5. LinkedOmics Analysis

LinkedOmics (http://www.linkedomics.org/login.php) on 1 April 2024, a public online analysis tool, contains cancer-associated multidimensional datasets derived from all 32 TCGA types [38]. First, we selected the “TCGA_BRCA” cancer cohort and the following parameters: data type, “mutation”; attribute, “*CDH1*”; sample dataset, “infiltrating lobular carcinoma”; target dataset, “RNA-seq”, “reverse phase protein array (RPPA)”, “methylation”, and “miRNA-seq”. The statistical method used was the Wilcoxon test. *CDH1* mRNA, protein, and methylation levels were compared between the *CDH1*-mutant and non-mutant groups. For miRNAs, hsa-mir-676 was most highly expressed in the *CDH1* non-mutant group; therefore, its expression was compared between the two groups. Moreover, enrichment analysis using GO annotations, GO (biological process), GO analysis (cellular component), and GO (molecular function) was performed using the Linkinterpreter for RNA-seq data [select tool: GSEA; rank criteria: *p*-value; simulations: 500 times]. Differences were considered significant when both the *p*-value and FDR were <0.05.

### 4.6. Statistical Analysis

The clinicopathological and intrinsic subtype differences between *CDH1*-altered and -unaltered ILC were analyzed using the chi-squared test. The TMB between *CDH1*-altered and -unaltered samples was analyzed using the Mann–Whitney U test. Chi-squared tests were performed to determine the frequency of ILC-related mutations. Statistical significance was set at *p* < 0.05 and FDR < 0.05. All statistical analyses were performed using EZR software (version 1.55) [39].

## 5. Conclusions

In summary, *CDH1*-unaltered ILC was more frequently associated with the CL subtype, resulting in a different subtype distribution compared to *CDH1*-altered ILC, which led to significant differences in associated gene mutations. Interestingly, the LinkedOmics results revealed no significant difference in E-cadherin protein levels between *CDH1*-mutated and *CDH1* non-mutated ILC, demonstrating that E-cadherin expression is impaired in *CDH1* non-mutated ILC. Considering that there were no differences in mRNA and methylation levels, post-translational modifications (such as aberrant glycosylation) and N-terminal-deficient E-cadherin production are the most plausible explanations for E-cadherin inactivation in *CDH1*-unaltered ILC, and this subject warrants further investigation.

## Figures and Tables

**Table 1:** Type and frequency of *CDH1* alterations.

Alteration Type	TCGA (n = 110)	METABRIC (n = 91)	Total (n = 201)
Mutation			
Missense, n (%)	4 (3.6)	9 (9.9)	13 (6.5)
In-frame, n (%)	4 (3.6)	1 (1.1)	5 (2.5)
Splice, n (%)	11 (10.0)	12 (13.2)	23 (11.4)
Truncating, n (%)	82 (74.5)	65 (71.4)	147 (73.1)
Homozygous deletion and mutation, n (%)	3 (2.7)	1 (1.1)	4 (2.0)
Homozygous deletion, n (%)	4 (3.6)	3 (3.3)	7 (3.5)
Structural variant, n (%)	2 (1.8)	0 (0)	2 (1.0)

TCGA, The Cancer Genome Atlas; METABRIC, Molecular Taxonomy of Breast Cancer International Consortium.

**Table 2:** Comparison of clinicopathological features between *CDH1*-altered and -unaltered ILC.

Clinicopathological Feature	*CDH1*-Altered ILC (n = 201)	*CDH1*-Unaltered ILC (n = 192)	*p*-Value
Age			
Mean	63.1	62.1	0.51
Minimum	37	27	
Maximum	90	71	
Site			
Left, n (%)	89 (50.0)	88 (55.3)	0.38
Right, n (%)	89 (50.0)	71 (44.7)	
N/A	23	33	
Menopausal status			
Pre, n (%)	20 (11.9)	30 (19.9)	0.07
Post, n (%)	148 (88.1)	121 (80.1)	
N/A	33	41	
T factor			
T1, n (%)	42 (23.6)	39 (25.2)	0.99
T2, n (%)	92 (51.7)	79 (51.0)	
T3, n (%)	43 (24.2)	36 (23.2)	
T4, n (%)	1 (0.6)	1 (0.6)	
N/A	23	37	
N factor			
N0, n (%)	58 (53.2)	36 (40.0)	0.07
N1, n (%)	22 (20.2)	33 (36.7)	
N2, n (%)	10 (9.2)	8 (8.9)	
N3, n (%)	19 (17.4)	13 (14.4)	
N/A	92	102	
Tumor grade			
Grade 1, n (%)	6 (7.1)	11 (11.5)	0.38
Grade 2, n (%)	54 (63.5)	52 (54.2)	
Grade 3, n (%)	25 (29.4)	33 (34.4)	
N/A	116	96	
TMB			
Mean	5.6	4.2	0.05
SD	9.5	44.8	
Median	2.6	2.5	
Minimum	0	0	
Maximum	103.1	28.8	
TMB-low (<10) (%)	169 (84.1)	159 (88.3)	0.29
TMB-high (≥10) (%)	32 (15.9)	21 (11.7)	
N/A	0	12	

ILC, invasive lobular carcinoma; TMB, tumor mutation burden; SD, standard deviation; N/A, not applicable.

**Table 3:** Comparison of molecular subtypes between *CDH1*-altered and -unaltered ILC.

	TCGA			METABRIC		
Subtype	*CDH1*-Altered (n = 110)	*CDH1*-Unaltered (n = 91)	*p*-Value	*CDH1*-Altered (n = 91)	*CDH1*-Unaltered (n = 101)	*p*-Value
Luminal A, n (%)	95 (88.8)	39 (70.9)	0.06	38 (53.5)	26 (35.1)	<0.05
Luminal B, n (%)	4 (3.7)	7 (12.7)		14 (19.7)	8 (10.8)	
Her2, n (%)	2 (1.9)	1 (1.8)		6 (8.6)	8 (10.8)	
Basal, n (%)	1 (0.9)	1 (1.8)		0 (0)	4 (5.4)	
Normal-like, n (%)	5 (4.7)	7 (12.7)		11 (15.5)	15 (20.3)	
Claudin-low, n (%)	Blank	Blank		2 (2.8)	13 (17.6)	
N/A	3	36		20	27	

Her2, Human epidermal growth factor receptor 2; TCGA, The Cancer Genome Atlas; METABRIC, Molecular Taxonomy of Breast Cancer International Consortium; ILC, invasive lobular carcinoma; N/A, not applicable.

## Data Availability

The datasets generated and/or analyzed in this study are available from the corresponding author on reasonable request.

## Supplementary Materials

The following supporting information can be downloaded at https://www.mdpi.com/article/10.3390/ijms25168961/s1.
